# Exophytic Papillary Thyroid Carcinoma With Anaplastic Transformation in a Cervical Lymph Node: A Case Report and Review of the Literature

**DOI:** 10.7759/cureus.116776

**Published:** 2026-09-23

**Authors:** Israel Garcia Avilán, Oscar R Garcia, Jesus Delgado, Brandon Lopez Alanis, Flor Ortiz Villeda

**Affiliations:** 1 General Surgery, Hospital Universitario "Dr. José Eleuterio González", Monterrey, MEX; 2 Oncology, Hospital Universitario "Dr. José Eleuterio González", Monterrey, MEX; 3 Pathology, Hospital Universitario "Dr. José Eleuterio González", Monterrey, MEX

**Keywords:** anaplastic transformation, cervical lymph node metastasis, exophytic thyroid carcinoma, extrathyroidal extension, papillary thyroid carcinoma, tall-cell variant

## Abstract

Papillary thyroid carcinoma (PTC) is the most common malignant neoplasm of the thyroid and generally follows an indolent clinical course with favorable long-term survival. However, a subset of tumors may develop aggressive histological and locoregional features, including tall-cell morphology, extensive extrathyroidal extension, and anaplastic transformation. We report an unusual presentation of PTC characterized by massive exophytic cervical growth and anaplastic transformation within a metastatic cervical lymph node. A 76-year-old man presented with a progressively enlarging right lateral cervical mass over a two-year history, accompanied by hoarseness, dysphonia, and dyspnea on moderate exertion. Physical examination revealed a 15 × 15 cm exophytic cervical mass with overlying skin ulceration. Computed tomography demonstrated extensive right cervical lymphadenopathy with central necrosis, compression of the ipsilateral internal jugular vein, airway displacement, and a heterogeneous right thyroid lobe mass with internal calcifications and poorly defined margins. The patient underwent total thyroidectomy and right radical neck dissection. Intraoperatively, an extensive cervical nodal conglomerate with infiltration of the sternocleidomastoid muscle and involvement of the thyroid cartilage was identified. Histopathological examination demonstrated papillary thyroid carcinoma of the tall-cell subtype with anaplastic transformation within cervical lymph node metastasis. This case highlights an exceptionally aggressive locoregional presentation of PTC and emphasizes the importance of comprehensive clinical, radiological, surgical, and histopathological assessment in patients presenting with bulky cervical disease. Recognition of aggressive histological features and anaplastic transformation is essential for appropriate multidisciplinary management and prognostic assessment.

## Introduction

Papillary thyroid carcinoma (PTC) is the most common malignant neoplasm of the thyroid and generally has a favorable prognosis. Most patients present with an asymptomatic thyroid nodule or cervical lymphadenopathy. Although regional lymph node metastases are relatively common, extensive locoregional disease is less frequent [1].

Extrathyroidal extension (ETE) is an important prognostic feature of differentiated thyroid carcinoma and may range from microscopic involvement of perithyroidal tissues to gross invasion of surrounding structures. Extensive macroscopic ETE involving the cervical soft tissues, skin, muscles, airway structures, or laryngeal framework is uncommon and is associated with more aggressive disease and less favorable oncological outcomes [2].

The tall-cell subtype of PTC is an aggressive histological variant characterized by distinctive cytomorphological features and a higher propensity for extrathyroidal extension, lymph node metastasis, and recurrent disease [1,3]. In addition, differentiated thyroid carcinomas may rarely undergo anaplastic transformation, resulting in a markedly more aggressive phenotype and poorer prognosis. Anaplastic transformation may occur within the primary thyroid tumor or within metastatic lymph node deposits and represents an uncommon but clinically significant event [4-6].

We present an unusual case of a 76-year-old man with a massive exophytic cervical presentation of PTC, extensive locoregional involvement, and anaplastic transformation identified in both the primary tumor and a cervical lymph node. A review of the relevant literature is also provided to contextualize the clinical, pathological, and surgical characteristics of this rare presentation.

## Case presentation

A 76-year-old man presented with a progressively enlarging right lateral cervical mass with a two-year history. The patient reported progressive hoarseness, dysphonia, and dyspnea on moderate exertion. No previous history of thyroid disease or cervical irradiation was documented.

Prior to surgery, flexible nasoendoscopy was performed to evaluate vocal cord mobility, which demonstrated paralysis of the right vocal cord in the adducted position, with asymmetric vocal fold movement during phonation. The true vocal folds were otherwise intact, symmetrical, and eutrophic. The left vocal fold demonstrated adequate abduction and adduction during phonation and during maneuvers, with no residual glottic gap. No aspiration or penetration of secretions was observed.

Physical examination revealed a large exophytic mass occupying the right lateral neck, measuring approximately 15 × 15 cm. The lesion was firm, irregular, partially fixed to the deep planes, and associated with overlying skin ulceration (Figure 1). No active bleeding was identified at the time of initial evaluation. Marked cervical asymmetry, local inflammatory changes, and distortion of the cervical contour were present.

**Figure 1:**
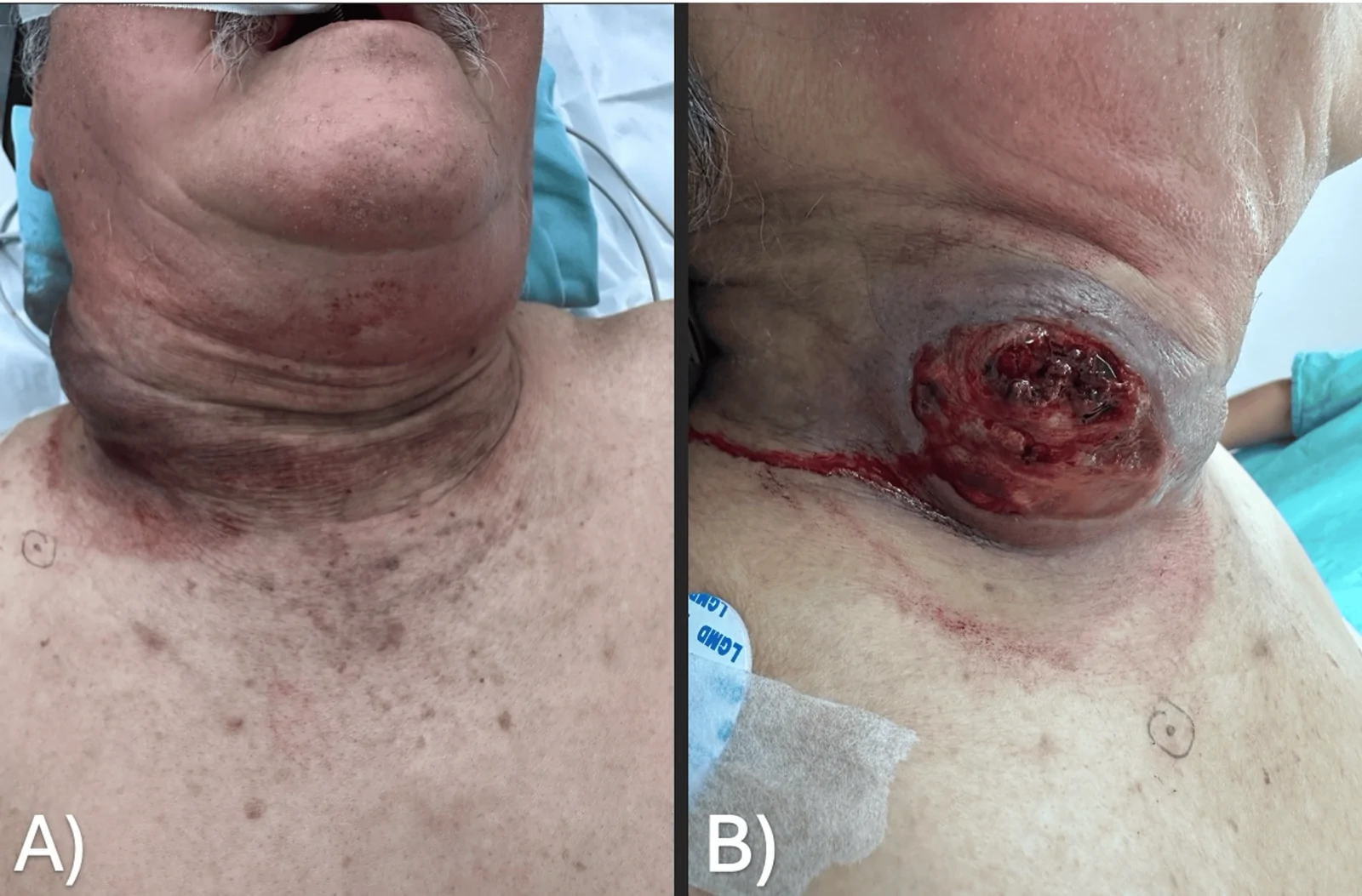
Clinical presentation. (A) Anterior view of the patient's neck, showing an enlargement of its right side. (B) Large exophytic mass involving the right lateral neck, measuring approximately 15 × 15 cm, with center-overlying skin ulceration.

Contrast-enhanced computed tomography (CT) of the neck demonstrated multiple right cervical lymph nodes involving levels IVA and VIB. The largest lesion consisted of a conglomerate nodal mass with central hypodense areas suggestive of necrosis, measuring approximately 6.1 × 6.4 × 6.9 cm in the anteroposterior, craniocaudal, and laterolateral dimensions, respectively. The lesion was in close contact with the sternocleidomastoid muscle and the common carotid artery and caused compression of the ipsilateral internal jugular vein and mild displacement of the airway. An associated subcutaneous collection containing internal gas locules measured approximately 4.1 × 1.1 × 4.5 cm; these gas locules were considered attributable to the overlying skin ulceration and associated localized superinfection of the subcutaneous collection.

A heterogeneous mass arising from the right thyroid lobe, measuring approximately 3.9 × 2.8 × 4.2 cm, was also identified. The lesion demonstrated poorly defined margins and internal calcifications and was associated with multiple adjacent cervical lymph nodes, several of which demonstrated central necrosis. A heterogeneous nodule was additionally identified in the left thyroid lobe (Figure 2).

**Figure 2:**
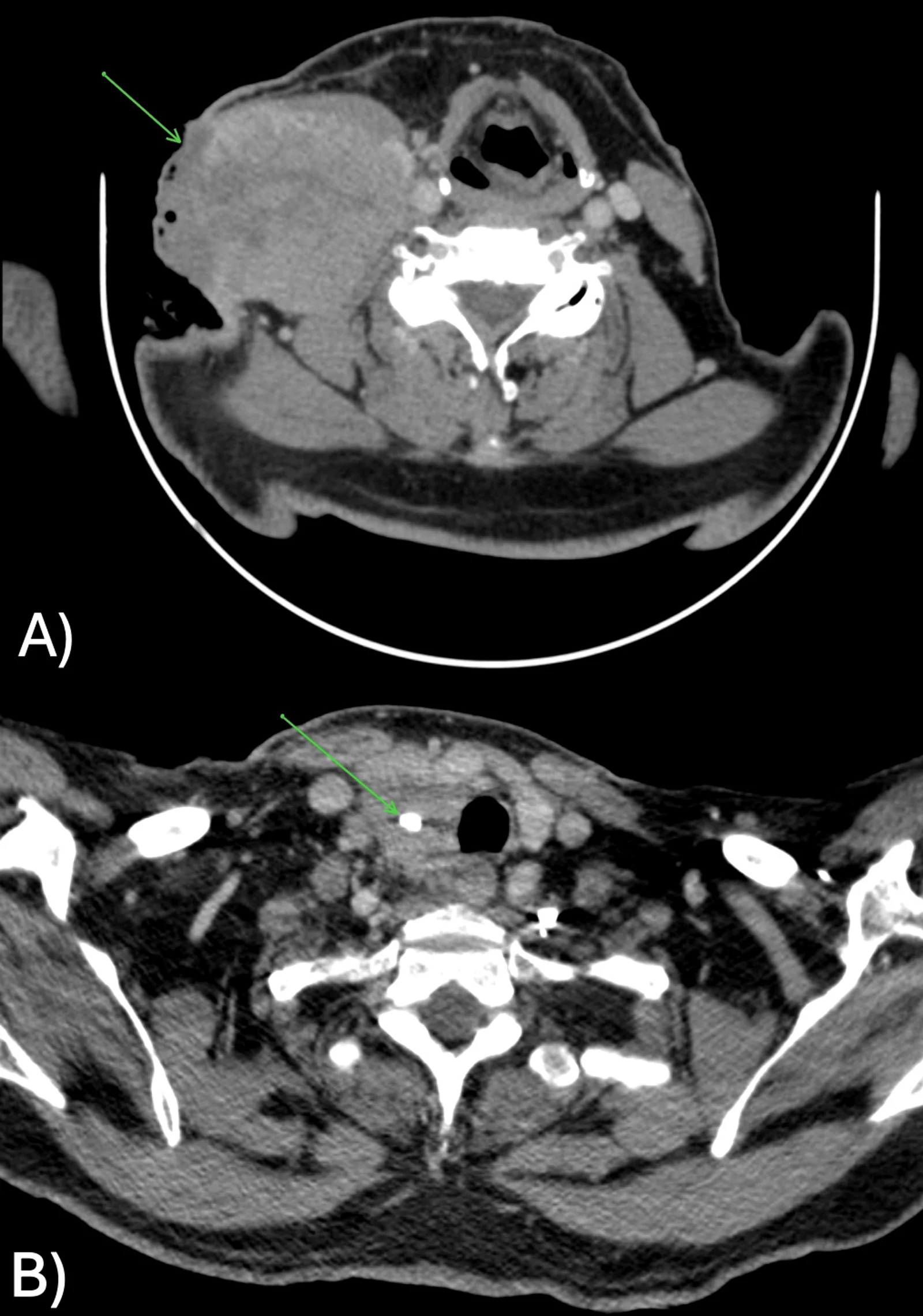
Computed tomography findings. (A) Axial contrast-enhanced computed tomography demonstrating a heterogeneous right thyroid lesion with internal calcification and associated cervical lymphadenopathy. (B) The arrow points to a large right cervical nodal conglomerate with heterogeneous attenuation and central necrotic areas.

Given the extent of disease and the clinical and radiological findings, the patient underwent total thyroidectomy with right radical neck dissection. Intraoperatively, a large cervical nodal conglomerate measuring approximately 10 × 10 cm and involving cervical levels IIA, III, and IV was identified. The mass infiltrated the sternocleidomastoid muscle and demonstrated extensive skin ulceration with friable and hemorrhagic tumor tissue.

The carotid sheath structures were preserved. The left recurrent laryngeal nerve was identified and preserved. A tumor arising from the right thyroid lobe with involvement of the thyroid cartilage was identified. Shaving of the involved thyroid cartilage was performed; final pathological assessment demonstrated microscopic residual disease at this level, corresponding to an R1 resection. The right recurrent laryngeal nerve could not be preserved because of intimate tumor involvement. Multiple enlarged lymph nodes from the central compartment were resected and submitted for histopathological examination. Figure 3 features intraoperative images and the surgical piece.

**Figure 3:**
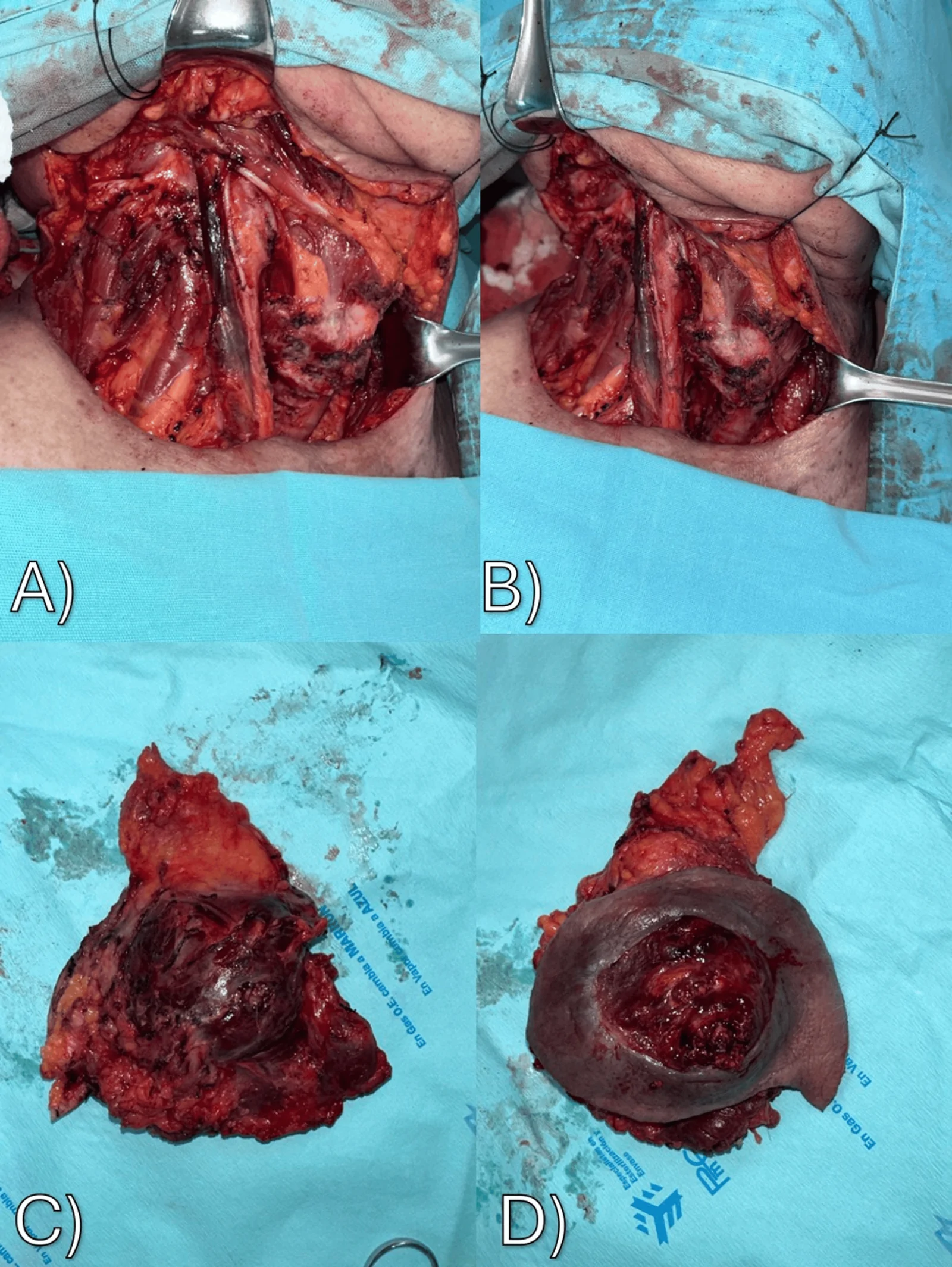
Intraoperative and postoperative findings. (A) Anterior view of the neck following resection of the cervical nodal conglomerate and total thyroidectomy. (B) Right lateral cervical view demonstrating dissection of the sternocleidomastoid muscle because of its intimate relationship with the tumor. The right carotid sheath and trachea remained intact. (C) Internal surface of the surgical specimen demonstrating a large lymph node with attached muscle fibers from the right sternocleidomastoid muscle. (D) External surface of the surgical specimen demonstrating central ulceration.

Histopathological examination confirmed papillary thyroid carcinoma of the tall-cell subtype in the thyroid specimen. A focal abrupt transition between the papillary-patterned tumor and an adjacent anaplastic component was identified. The anaplastic component demonstrated a solid growth pattern, marked nuclear pleomorphism, open chromatin, and numerous apoptotic bodies. Immunohistochemical analysis demonstrated loss of thyroglobulin expression within the anaplastic component, while BRAF V600E expression was retained in both the differentiated and anaplastic components.

Additional pathological findings, shown in Figure 4, included multiple metastatic cervical lymph nodes and perineural invasion. Anaplastic features were also identified morphologically within the metastatic cervical lymph node, in addition to the primary tumor, although Figure 4 did not include a dedicated photomicrograph capturing this finding within the lymph node. Overall, the findings were consistent with papillary thyroid carcinoma of the tall-cell subtype with anaplastic transformation involving both the primary tumor and a cervical lymph node metastasis.

**Figure 4:**
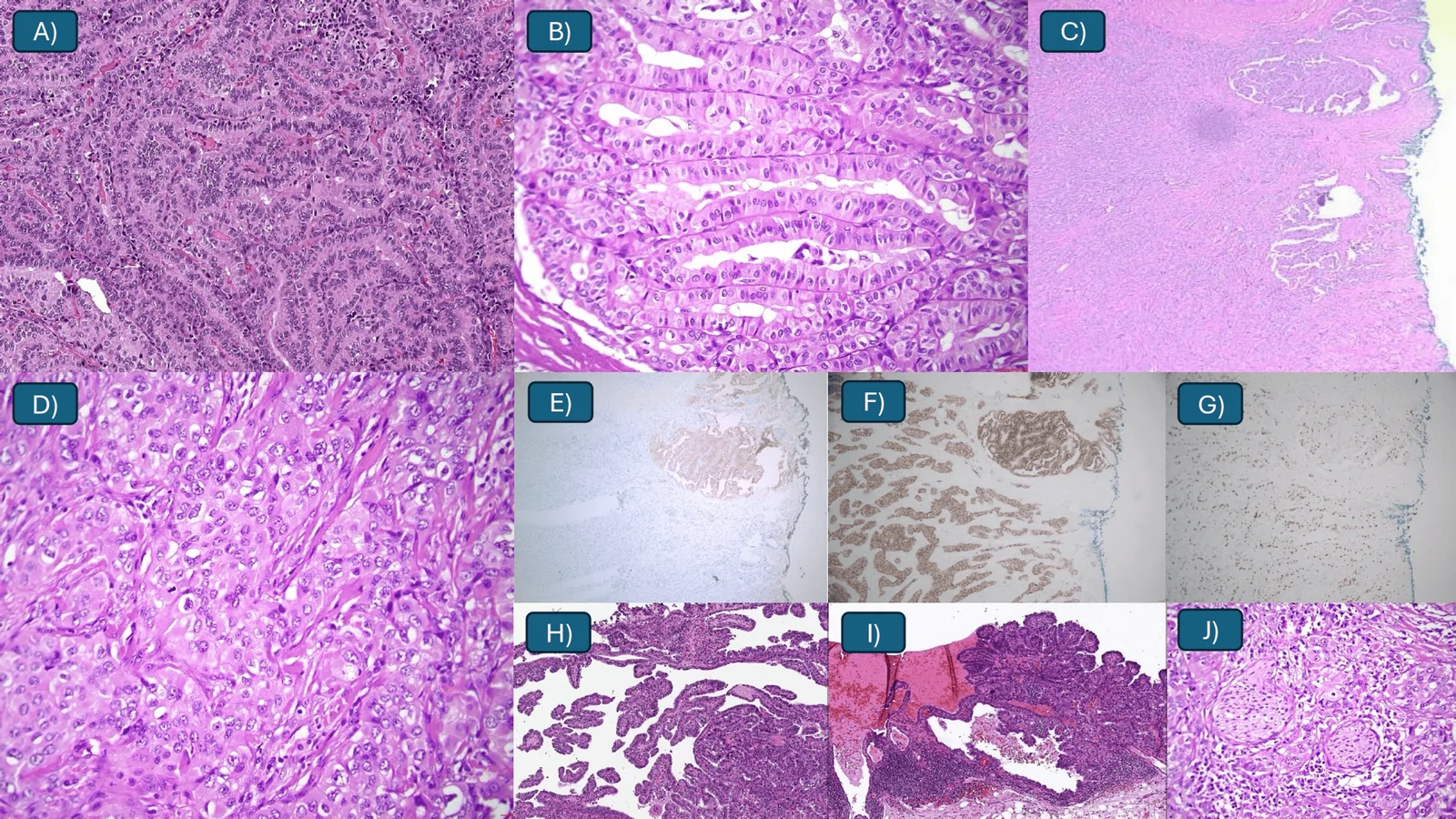
Histopathological and immunohistochemical findings. (A) Trabecular architecture composed of closely packed, back-to-back glands (10×). (B) Tall neoplastic cells measuring approximately two to three times their width, with well-defined cytoplasmic borders, eosinophilic cytoplasm, and nuclear features characteristic of papillary thyroid carcinoma (20×). (C) Abrupt transition between a papillary-patterned neoplasm and an adjacent anaplastic component (4×). (D) Anaplastic component demonstrating solid growth, marked nuclear pleomorphism, open chromatin, and numerous apoptotic bodies (20×). (E) Loss of thyroglobulin expression in the anaplastic component. (F) Retained BRAF V600E in both the differentiated and anaplastic components. (G) Ki-67 proliferation index of approximately 20%. (H) Area demonstrating conventional papillary growth. (I) Metastatic cervical lymph node involvement. (J) Perineural invasion.

At low magnification (10×), the tumor demonstrated a trabecular architecture composed of closely packed, back-to-back glands (Figure 4A). At higher magnification (20×), the neoplastic cells were approximately two to three times taller than they were wide, imparting the characteristic appearance of tall, rectangular cells. These cells exhibited well-defined cytoplasmic borders, eosinophilic cytoplasm, and nuclear features characteristic of papillary thyroid carcinoma (Figure 4B).

At low magnification (4×), an abrupt transition was observed between a papillary-patterned neoplasm and an adjacent anaplastic component (Figure 4C). The anaplastic component displayed a solid growth pattern with marked nuclear pleomorphism, open chromatin, and numerous apoptotic bodies (20×) (Figure 4D). Immunohistochemical studies demonstrated loss of thyroglobulin expression in the anaplastic component (Figure 4E), whereas BRAF V600E expression was retained in both the differentiated and anaplastic areas (Figure 4F). The Ki-67 proliferation index was approximately 20% specifically within the anaplastic component (Figure 4G). Areas demonstrating a conventional papillary growth pattern were also identified (Figure 4H). Additional findings included multiple metastatic lymph nodes (Figure 4I) and perineural invasion (Figure 4J).

Systemic staging with contrast-enhanced computed tomography of the chest and abdomen did not identify evidence of distant metastatic disease, and no other relevant findings were noted.

The patient had a 10-day postoperative hospital stay, with intensive monitoring during the initial 48 hours following sacrifice of the right recurrent laryngeal nerve. He was treated with prophylactic antibiotics and oral calcium supplementation, and was discharged with a referral to medical and radiation oncology for adjuvant therapy. A chemotherapy regimen had not yet been established at the time of this report; this decision was postponed by the oncology team following an interim febrile episode, pending re-evaluation at a subsequent follow-up appointment.

## Discussion

Papillary thyroid carcinoma is a differentiated thyroid malignancy that generally has a favorable prognosis; however, several clinicopathological characteristics are associated with more aggressive disease and poorer outcomes, including advanced age, male sex, increased tumor size, multifocality, locoregional extension, lymph node or distant metastasis, and extrathyroidal extension [1,2]. Although microscopic extrathyroidal extension may occur in differentiated thyroid carcinoma, massive exophytic cervical growth with skin ulceration and extensive soft-tissue involvement represents an unusual clinical manifestation.

Anaplastic transformation of differentiated thyroid carcinoma is uncommon and may occur within the primary thyroid tumor or, less frequently, within metastatic lymph node deposits [3-5]. The present case is notable for the coexistence of extensive exophytic cervical disease and anaplastic transformation identified in both the primary thyroid tumor and a metastatic cervical lymph node.

Several high-risk features were present in this patient, including advanced age, a large exophytic cervical mass, extensive lateral cervical lymphadenopathy, necrotic nodal disease, skin ulceration, airway displacement, thyroid cartilage involvement, and tall-cell histology. Particularly significant was the identification of anaplastic transformation involving both the primary tumor and metastatic cervical lymph node tissue, representing an uncommon manifestation of tumor progression.

The tall-cell subtype of PTC has been associated with more aggressive clinicopathological characteristics than conventional PTC, including increased rates of extrathyroidal extension, lymph node metastasis, and recurrent disease [1,3]. Therefore, the presence of tall-cell morphology in a tumor with extensive locoregional disease represents an important adverse pathological feature.

Anaplastic transformation is characterized by loss of differentiated tumor characteristics and acquisition of a highly aggressive phenotype. Previous reports have described anaplastic transformation of PTC within both primary thyroid tumors and metastatic sites [3-6]. Although the molecular mechanisms underlying this transformation are not completely understood, alterations in molecular pathways associated with tumor progression have been implicated. Increased proliferative activity and abnormalities involving tumor suppressor pathways may also contribute to anaplastic transformation [5].

The identification of specific histological patterns in metastatic lymph nodes may be clinically relevant. Solid or insular growth and hobnail cell features have been described as potential morphological indicators of subsequent anaplastic transformation in nodal disease [6]. In the present case, the histopathological examination demonstrated an abrupt transition between the differentiated papillary component and an anaplastic component within the primary tumor, and anaplastic features were also identified morphologically within the metastatic lymph node, supporting the diagnosis of anaplastic transformation rather than a separate synchronous malignancy.

Table 1 summarizes the clinicopathological features of the present case in comparison with previously reported cases of anaplastic transformation involving cervical lymph node metastases of PTC [3-6].

**Table 1:** Comparison with previously reported cases of anaplastic transformation involving cervical lymph node metastases of PTC. Note: For Kovacevic et al. [4] and Song et al. [5], detailed treatment and outcome data beyond those summarized above were not available in the accessible published sources. PTC: papillary thyroid carcinoma.

Reference	Year	Age/sex	Site of anaplastic transformation	Treatment	Outcome
Present case	2026	76, M	Primary thyroid tumor and cervical lymph node metastasis	Total thyroidectomy + right radical neck dissection (R1); referred for adjuvant therapy	Adjuvant therapy pending at time of report
Benedict and Costa [3]	2016	Not reported (diagnosed at autopsy)	Multiple distant metastatic sites (lung, pleura, pericardium, adrenal gland, liver, mediastinal/abdominal/hilar lymph nodes)	None (postmortem diagnosis)	Diagnosis made after death
Kovacevic et al. [4]	2025	66, F	Cervical lymph node metastasis (primary tumor regressed/sclerotic)	Total thyroidectomy + metastasectomy	Not detailed in available source
Song et al. [5]	2020	85, M	Cervical lymph node metastasis (coexisting with PTC; thyroid showed PTC only)	Surgical resection (not detailed in available source)	Not detailed in available source
Odate et al. [6]	2021	Case series (n=10)	Regional lymph node recurrence	Variable (retrospective series)	Study of histological predictors, not a single-patient outcome

The differential diagnosis of a large exophytic lateral cervical mass is broad and includes branchial cleft cysts, metastatic squamous cell carcinoma, lymphoma, soft-tissue sarcoma, infected branchial cleft cyst, and suppurative cervical lymphadenopathy. Cystic cervical masses are frequently associated with benign developmental lesions, whereas metastatic squamous cell carcinoma represents an important malignant differential diagnosis in older adults. Differentiated thyroid carcinoma presenting as a massive exophytic cervical lesion is uncommon [7]. In the present case, the identification of a suspicious thyroid lesion associated with extensive necrotic cervical lymphadenopathy provided an important diagnostic clue toward an advanced thyroid malignancy.

Imaging plays an essential role in defining the extent of locoregional disease and identifying involvement of vascular, muscular, airway, and laryngeal structures. In patients with gross extrathyroidal extension, detailed preoperative imaging is particularly important for determining the feasibility and extent of surgical resection. In this case, CT demonstrated extensive cervical nodal disease, compression of the ipsilateral internal jugular vein, airway displacement, and a heterogeneous thyroid lesion with internal calcifications. Systemic staging with chest and abdominal CT excluded distant metastatic disease, which is an important consideration for surgical decision-making and prognosis in patients with extensive locoregional involvement.

Surgical management of advanced locoregional PTC requires individualized assessment of tumor extent and the relationship between the tumor and adjacent anatomical structures. In selected patients, thyroidectomy combined with therapeutic neck dissection and resection of involved adjacent structures may provide locoregional control. However, extensive invasion may preclude complete microscopic resection, as occurred in the present case, in which microscopic residual disease remained at the involved thyroid cartilage.

The presence of an R1 resection at the level of the thyroid cartilage, together with retained BRAF V600E expression in both the differentiated and anaplastic components, carries important therapeutic implications. BRAF/mitogen-activated protein kinase kinase (MEK) inhibitor combinations, such as dabrafenib and trametinib, have demonstrated meaningful responses in BRAF V600E-mutated anaplastic thyroid carcinoma and may be considered as part of a multidisciplinary treatment strategy in this setting. External beam radiotherapy may also be considered to improve locoregional control in patients with microscopic or gross residual disease. In the present case, definitive decisions regarding the role of radiotherapy and systemic or targeted therapy were deferred to multidisciplinary oncology evaluation.

The unusual combination of massive exophytic growth, extensive cervical nodal disease, tall-cell histology, and anaplastic transformation underscores the heterogeneous biological behavior of PTC. Although PTC is generally associated with favorable outcomes, the presence of aggressive histological variants and anaplastic transformation may substantially alter prognosis and therapeutic considerations. Recognition of these features is therefore important for multidisciplinary treatment planning and appropriate patient counseling.

## Conclusions

Exophytic papillary thyroid carcinoma with extensive cervical involvement represents an uncommon manifestation of advanced thyroid malignancy. The present case is particularly notable for the coexistence of massive exophytic growth, extensive locoregional disease, tall-cell histology, and anaplastic transformation involving both the primary thyroid tumor and metastatic cervical lymph node tissue.

A thorough clinical examination and cross-sectional imaging, including systemic staging to exclude distant metastatic disease, are essential for defining disease extent and guiding surgical planning in patients presenting with bulky cervical masses. Histopathological evaluation remains critical for identifying aggressive histological variants and anaplastic transformation, which may significantly influence prognosis and subsequent treatment. Recognition of these high-risk features should prompt multidisciplinary management involving endocrine surgery, head and neck surgery, pathology, radiology, endocrinology, and oncology to establish an individualized therapeutic strategy, including consideration of adjuvant radiotherapy and BRAF/MEK-targeted therapy where appropriate.
